# Educational interventions aimed at improving knowledge of delirium among nursing home staff—a realist review

**DOI:** 10.1186/s12877-024-05213-9

**Published:** 2024-07-25

**Authors:** Vincent Molitor, Theresa Sophie Busse, Chantal Giehl, Romy Lauer, Ina Carola Otte, Horst Christian Vollmar, Petra Thürmann, Bernhard Holle, Rebecca Palm

**Affiliations:** 1 Faculty of Health, School of Nursing Science, Witten Herdecke University, Witten, Germany; 2 Institute of General Practice and Family Medicine (AM RUB), Medical Faculty, Ruhr University Bochum, Bochum, Germany; 3 Faculty of Health, School of Medicine, Chair of Clinical Pharmacology, Witten Herdecke University, Witten, Germany; 4 Philipp Klee-Institute of Clinical Pharmacology, Helios University Hospital Wuppertal, Wuppertal, Germany; 5 German Center for, Deutsches Zentrum fur Neurodegenerative Erkrankungen (DZNE) Standort Witten, Witten, Germany; 6 Department of Geriatric Medicine, Marien Hospital Herne, Ruhr University Bochum, Herne, Germany; 7 School VI -School of Medicine and Health Sciences, Carl von Ossietzky Universität, Oldenburg, Germany

**Keywords:** Nurses, Interprofessional Work, Interprofessional Education, Education, Realist Review, Nursing Homes, Delirium, Cognitive Dysfunction, Health Personnel, Communication

## Abstract

**Background:**

Delirium is a neuropathological syndrome that is characterised by fluctuating impairments in attention, cognitive performance, and consciousness. Since delirium represents a medical emergency, it can be associated with adverse clinical and economic outcomes. Although nursing home residents face a high risk of developing delirium, health care professionals in this field appear to have limited knowledge of delirium despite the critical role they play in the prevention, diagnosis, and treatment of delirium in nursing homes.

**Objective:**

The purpose of this realist review is to develop an initial programme theory with the goal of understanding how, why, and under what circumstances educational interventions can improve the delirium-specific knowledge of health care professionals in nursing homes.

**Methods:**

This realist review was conducted in accordance with the RAMESES (Realist And Meta-narrative Evidence Synthesis: and Evolving Standards) guidelines and includes the following steps: (1) search strategy and literature review; (2) study selection and assessment; (3) data extraction; (4) data synthesis; and (5) development of an initial programme theory. It also included stakeholder discussions with health care professionals recruited from nursing home care, which focused on their experiences with delirium.

**Results:**

From a set of 1703 initially identified publications, ten publications were included in this realist review. Based on these publications, context-mechanism-outcome configurations were developed; these configurations pertained to (1) management support, (2) cognitive impairments among residents, (3) familiarity with residents, (4) participatory intervention development, (5) practical application, (6) case scenarios, (7) support from experts and (8) relevance of communication.

**Conclusions:**

Educational interventions aimed at improving the delirium-specific knowledge of health care professionals should feature methodological diversity if they are to enhance health care professionals’ interest in delirium and highlight the fundamental contributions they make to the prevention, diagnosis, and treatment of delirium. Educational interventions should also take into account the multidimensional contextual factors that can have massive impacts on the relevant mode of action as well as the responses of health care professionals in nursing homes. The identification of delirium in residents is a fundamental responsibility for nursing home staff.

**Trial registration:**

This review has been registered at Open Science Framework 10.17605/OSF.IO/6ZKM3

**Supplementary Information:**

The online version contains supplementary material available at 10.1186/s12877-024-05213-9.

## Background

Delirium involves the neuropathological impairment of attention, awareness, and cognitive functions. It is characterised by a fluctuating course and can vary in intensity and severity throughout the day [1]. Delirium is considered to constitute a medical emergency because the prognosis is negative if it is not detected and treated early [2]. The causes of delirium may include existing medical conditions such as infection, dehydration, or substance intoxication as well as the effects of pharmacotherapy [3]. Delirium can present with different motor subtypes, where hypoactive delirium is characterised predominantly by somnolence and hyperactive delirium is best described by agitation and possibly aggression. Mixed forms of delirium are often observed, in which the symptoms change throughout the day [4].

### Delirium in nursing homes

Nursing home residents face the risk of delirium due to various predisposing risk factors, including older age, neurodegenerative diseases such as dementia or Parkinson’s disease, and interactions among the many medications they take [5]. Prevalence estimates of the occurrence of delirium in nursing homes have varied from 1.4% to 70% [6]. No precise figures are available in the context of Germany. The reasons for this wide variation include different study designs and populations as well as the different measurement tools used to diagnose delirium. Furthermore, the diagnosis of delirium in the presence of dementia, which is known as delirium superimposed on dementia (DSD), is particularly challenging as it is partly similar in symptoms to dementia without delirium [7]. Another reason could be the variety of different definitions of nursing homes. These facilities differ from settings in which individuals are supported by outpatient services in their own homes and are instead places at which on-site nursing support is available 24 h per day, seven days per week [8].

Among nursing home residents, delirium is also associated with a variety of negative outcomes, including hospitalisations and increased mortality [9]. In addition, delirium can be linked to functional decline in nursing home residents [10]. For example, delirium and dementia have been shown to exhibit a complex interrelationship. Individuals who develop delirium are more likely to develop dementia at a later point and vice versa [11]. The economic burden of delirium, which results in prolonged hospital stays and loss of function, should not be neglected [12, 13].

### Lack of delirium-specific knowledge

Although delirium is one of the most common and serious complications of institutionalisation, it is often not recognised by nurses [14]. Nevertheless, prevention and early detection are the most important components of delirium care. Nurses’ clinical judgements determine whether the general practitioners providing treatment to these patients are notified and whether measures for diagnosis and therapy are implemented. However, studies have indicated that the level of delirium-specific knowledge among nurses in nursing homes is rather low [15, 16]. The reasons for this lack of knowledge are diverse. Research on curricula and discussions with nursing educators have revealed that little to no weight is given to the topic of delirium, e.g., in generalist nursing training in Germany. This limitation also seems to apply to health care professionals in other countries [17].

An interdisciplinary statement issued by scientific societies at the European level demanded the structured anchoring of delirium-specific knowledge throughout the training of all health care professionals [18]. Several multicomponent interventions drawn from the acute hospital setting, including those aimed at providing education on delirium to health care professionals, have achieved positive results in the acute hospital setting [19]. However, these interventions are not easily transferable to the nursing home setting [20]. Therefore, the National Institute for Health and Care Excellence (NICE) called for the development of adapted multicomponent interventions that could be applied in different settings [21].

### Complexity of educational interventions

Educational interventions can additionally be described as complex because they aim to change behaviour based on several determinants, such as the acquisition of knowledge, and often consist of discrete components that interact with each other and are not linear [22]. The authors of the updated Medical Research Council framework recommended a theory-based evaluation of complex interventions [23]. Given that complex interventions in general are highly dependent on the social context in which they are situated, it is crucial to examine how, why, for whom, and under what circumstances educational interventions work. This focus is primarily associated with proponents of the realist review methodology, which is grounded in critical realism.

## Objectives

The aim of the realist review presented here is to understand how, why, and under what circumstances educational interventions improve the knowledge of health care professionals concerning the phenomenon of delirium in nursing homes. Based on the results of this study, we present an initial programme theory concerning educational interventions and how they work.

## Methods

A realist review, which was guided by the “Realist And Meta-narrative Evidence Synthesis: Evolving Standards” (RAMESES) guidelines [24], was conducted. The Preferred Reporting Items for Systematic Review and Meta-Analysis Protocols (PRISMA-P) checklist was also used and can be found in Appendix 1.

### Context-mechanism-outcome configurations

The goal of a realist review is to understand the relationships among the relevant context, mechanisms, and outcomes. Context refers to elements in the environment surrounding an intervention that influence the corresponding outcomes (e.g., demographic, geographic, or cultural norms or laws) [25]. Mechanisms are the resources that are offered by an intervention and the associated responses to those resources (e.g., motivation, self-efficacy, or responsibility) [26]. Outcomes are based on the interactions between context and mechanism, which are usually measurable and occur at the behavioural or systems level [27]. Accordingly, realistic review methodology can be viewed as a suitable method for identifying context-mechanism-outcome configurations (CMOcs), which then collectively constitute a programme theory.

### Steps in the realist review process

**Fig. 1:**
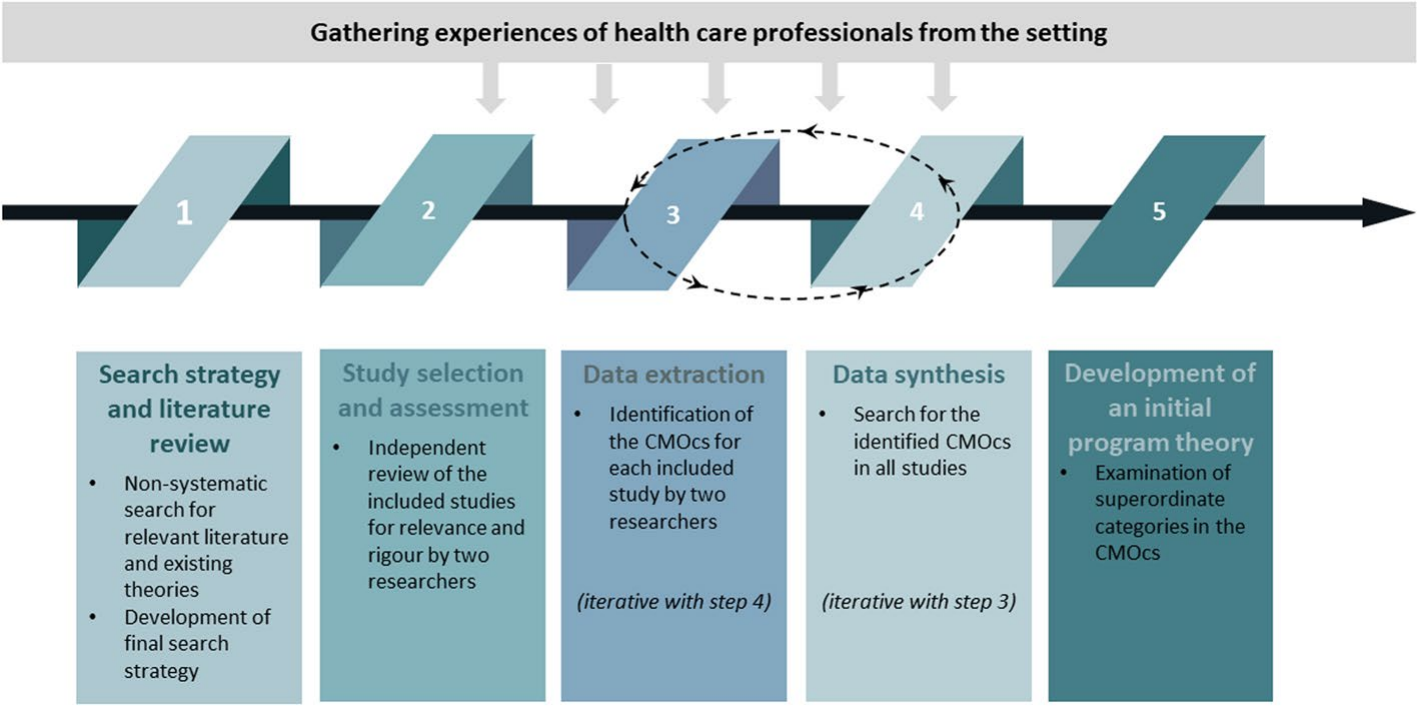
Steps involved in our realist review (authors’ own illustration)

The realist review included the following five steps: (1) search strategy and literature review; (2) study selection and assessment; (3) data extraction; (4) data synthesis; and (5) development of an initial programme theory. The sequence of steps can be found in Fig. 1.

#### Step (1) – Search strategy and literature review

**Fig. 2:**
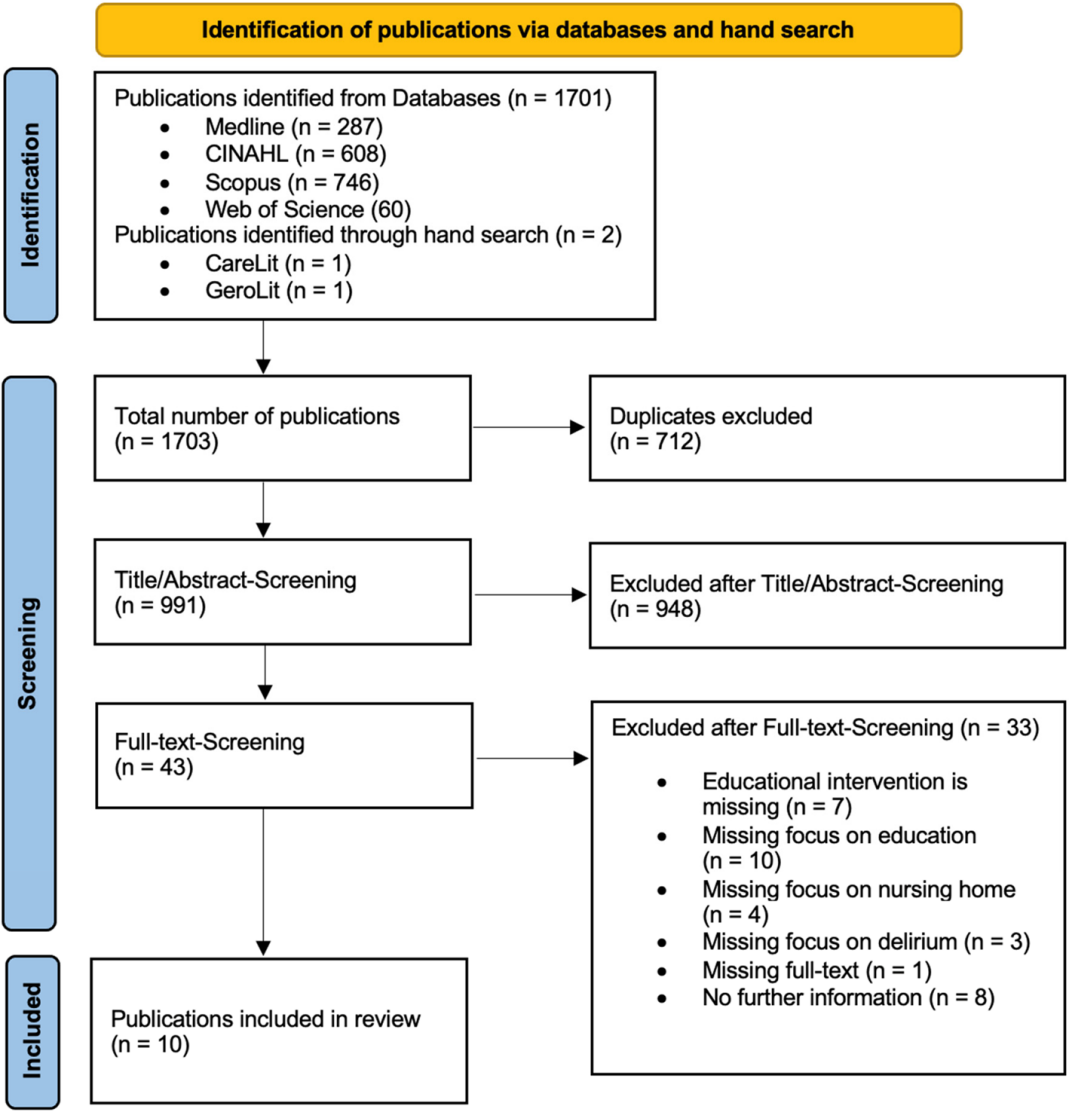
Flow chart for selection of the publications

To obtain an initial understanding, an exploratory and setting-independent literature search was conducted by VM. In addition, VM conducted a specific search to reveal learning theories that addressed the question of this realist review. The literature search illustrated in Fig. 2 was subsequently performed by reference to the Medline [PubMed], CINAHL [Ebsco], Scopus, and Web of Science databases. Additionally, the German databases GeroLit and CareLit were searched by hand based on the search string used for the rest of the search, as searching with complex search strings is not supported in these databases. The grey literature was searched for in a nonsystematic way. The literature search was conducted from October 2022 to January 2023 (by VM and CG).

The search strategy can be found in Appendix 2; furthermore, the inclusion and exclusion criteria developed for this search can be found in Appendix 3.

#### *Step 2* – *Study selection and assessment*

The title and abstract screening of the publications was performed independently by two researchers (VM, CG) using the platform Rayyan (Rayyan Systems, Inc., USA). These two researchers met regularly to discuss any disagreements regarding the inclusion or exclusion of individual publications. To reach consensus in two cases, RP was consulted to vote on whether the publications in question should be included or excluded. Full-text screening was conducted independently by VM and TSB. In this context, a high level of quality was ensured through detailed discussion of the criteria and objective. With regard to one publication, RP was consulted to discuss whether it should be included or excluded.

The selected publications were then subjected to quality appraisal (VM, TSB). The relevance and rigour of the data included in these publications were featured as key principles in the quality appraisal tool, which was developed by experts [24].

#### *Step 3* – *Data extraction*

After the quality appraisal, two researchers (VM, TSB) independently developed CMOcs for each included publication, which were compared and adjusted after completion and discussed among the research team (VM, TSB, RL, RP). This process resulted in 1–3 individual CMOcs per publication, which were discussed once again with the goal of enhancing the quality of this process (VM, RP, TSB). Subsequently, all included publications were transferred into the analysis software MAXQDA (VERBI – Software. Consult. Sozialforschung. GmbH, Germany) to search for all CMOcs with the goal of supporting a realist synthesis (VM, TSB).

#### *Step 4* – *Data synthesis*

The CMOcs that referred to individual publications were broken down into their components and compared to those associated with other identified publications. We searched for components of CMOcs that were identified in several publications and investigated whether different outcomes were achieved.

#### *Step 5* – *Development of an initial programme theory*

After comparing the CMOcs, an overarching analysis was conducted to identify the contextual factors that could impact the mechanism underlying educational interventions and the corresponding changes in outcomes. Finally, we categorised the CMOcs based on various important mechanisms and outcomes.

### Stakeholder involvement

Throughout the review process, we invited stakeholders experienced in geriatric care, four nurses and two general practitioners. These exchanges with the stakeholders encouraged us to focus on the aspects that were most relevant to them. We recruited these stakeholders through existing networks. RP, VM, TSB, and RL participated in the appointments. The exchange started with brief comments by the research team on delirium and the challenges of detecting it. This introduction was followed by questions about participants’ experiences recognising, dealing with and preventing delirium. Additionally, this study focused on interprofessional work in the context of delirium treatment and the existing delirium-specific knowledge of nursing staff. The conversations were logged, compared, and reviewed once again during the literature analysis.

### Protocol and registration

The protocol used for this realist review has been published elsewhere [28] and has been registered with the Open Science Framework (10.17605/OSF.IO/HTFU4).

### Changes from the submitted protocol during the review process

As part of the ongoing process of conducting the review, minor deviations from this were made to achieve the best possible results with regard to the research question. These changes are briefly described here.

The protocol indicated that the stakeholders would be involved only once, i.e., at the beginning of the realist review, with the goal of obtaining thematic input. During the course of the realist review, however, various stakeholders were involved throughout the entire review process to enable us to learn about and discuss their practical experiences with delirium in nursing homes.

The protocol described the aim of the realist review in terms of the development of an initial programme theory pertaining to nurses and general practitioners in nursing homes with the goal of developing educational interventions aimed at promoting knowledge about delirium. The literature revealed by the realist review focused almost exclusively on nurses, with the exception of one publication on multiprofessional teams. Therefore, the initial programme theory mostly addressed nurses/nursing assistants. In the protocol, the inclusion criteria focused on the reporting of delirium in any form alongside the fulfilment of the criteria stipulated in the International Classification of Disease-10 (ICD-10) or Diagnostic and Statistical Manual of Mental Disorders-V (DSM-V) classifications. Due to the strict limitations stipulated by these criteria and the focus of the research question on education rather than solely on nursing/medical findings in terms of prevalence, references to delirium per se were considered to be sufficient for the review.

### Researchers

The researchers who participated in the realist review had experience in health care/nursing/elderly care (RP, VM, TSB, BH) and had undergone graduate studies in nursing science (RP, VM, BH) and nursing (RP, VM, TSB, BH). In addition, researchers from the fields of health sciences/public health (TSB, CG, RL, IO, HCV) health economics (RL), gerontology (IO) and medicine (HCV, PT) contributed to this study. Furthermore, RP had experience with regard to the realist review methodology.

## Results

From the initial set of 1703 publications identified by the systematic literature search, evidence from ten publications was ultimately included. The publication selection process is described in Fig. 2.

The initial search resulted in a total of 1701 publications from four databases (Medline: *n* = 287, 17%; CINAHL: *n* = 608, 36%; Scopus: *n* = 746, 43%; Web of Science: *n* = 60, 4%). In addition, two records were identified through a manual search. No grey literature could be obtained. From the total of 1703 publications revealed by the initial search and the manual search, 991 (58%) publications remained after the elimination of duplicates.

Of these 991 publications, 948 (96%) were excluded during the title and abstract screening. The remaining 43 publications were subjected to full-text screening, which resulted in the inclusion of a total of ten publications in the evaluation. The studies described in these publications were conducted between 2008 and 2022 in the UK (*n* = 6 [29–34]), Canada (*n* = 2 [35, 36]), the USA (*n* = 1 [37]) and Korea (*n* = 1 [38]). The metadata of the studies included in this review can be found in Table 1.

**Table 1:** Metadata of the included publications

Author(s) and year	Educational intervention type	Nation	Study design	Outcomes	Relevance	Rigour
Brajtman et al. (2012) [35]	Self-learning module on end-of-life delirium and interprofessional teamwork, team objective structured clinical encounter (e.g., simulated team discussion and debriefing), and a didactic “theory burst”	Canada	Quantitative study using two questionnaires before and after the intervention in form of a pilot study	End-of-life delirium and perceptions of interprofessional competence improved	+ +	+
Featherstone et al. (2010) [29]	*Stop Delirium!*: Interactive teaching methods (including delirium practitioner, didactic sessions, delirium champions, and a tailored educational package for nursing homes). Provision of an enhanced educational package. A variety of interactive teaching methods were used over a 10-month period. Individuals who could champion the change were identified. Ownership was encouraged	UK	Mixed-methods study	A total of 91% of nursing home staff received education; 99.7% of HCPs claimed that the education was relevant; 97% of HCPs claimed that the time had been well spent	+ + +	+
Garden et al. (2016) [30]	Education programme was developed based on the *Stop Delirium!* material	UK	Quantitative study using questionnaires, correlation of data from care homes and hospital information department for comparison before and after intervention	Improvements in staff confidence in their ability to recognise, prevent, and manage factors associated with delirium as well as their knowledge of those factors	+	+
Jeong et al. (2022) [38]	Multifaceted and evidence-based delirium educational programme (including didactic training featuring delirium case scenarios as well as the provision of delirium materials and discussions to reflect delirium experiences)	Korea	Quantitative study using questionnaires as well as a comparison of ratings of delirium to compare before and after the intervention	Improvements in the participants’ delirium knowledge and confidence in their ability to delirium care and clinically detect delirium. No differences in patient outcomes (incidence of delirium) were observed	+ + +	+ +
Lewallen et al. (2019) [37]	Gerontologic nurse practitioner provided general delirium education sessions featuring case vignettes	USA	Quantitative study using a questionnaire to investigate interrater-reliability before and after an intervention	Improvement of delirium knowledge	+ +	+
Peacock et al. (2012) [31]	*Stop delirium!*	UK	Qualitative study using interviews for a secondary qualitative analysis	Triggers and knowledge, detection and observation, effect of the closest contact, changes in the management of care, use of communication and teamwork to overcome difficulties	+ +	+
Siddiqi et al. (2008) [33]	*Stop delirium!*	UK	Mixed-methods study using questionnaires and observations on the delivery of the intervention as well as data from records on the costs for a participatory design in form of a feasibility study	Time well spent, relevance to participants’ work, content appropriate to their needs	+ + +	+ +
Siddiqi et al. (2011) [34]	*Stop delirium!*	UK	Mixed-methods study using questionnaires, feedback forms, data from records, interviews and focus groups as well as logs before and after the intervention	Increased awareness of delirium and examples of changes in practice. Evidence supporting positive changes in personnel attitudes and practice and increases in personnel confidence in their ability to provide delirium care were observed after the intervention	+ + +	+ + +
Siddiqi et al. (2016) [32]	*Stop delirium!*	UK	Quantitative study using data from care home records and hospitals, tests and assessments for a feasibility cluster RCT	No improvements in incidence or prevalence were observed after the intervention	+ +	+ +
Voyer et al. (2014) [36]	Multicomponent programme featuring an educative component (PowerPoint). Based on all the information collected during the previous steps, the research team developed the following delirium prevention programme tools: a decision tree, an evaluation and intervention instruction manual, a delirium prevention toolkit, and PowerPoint presentations regarding the delirium prevention programme tools specifically for participating health care professionals (nurses, nursing assistants, and orderlies). The advisory committee and the long-term care clinical nurse specialist committee both provided input, which, alongside the clinical judgement of the research team members, contributed to tool selection and the decision-making process	Canada	Quantitative study using questionnaires for feasibility and acceptability in a participatory design study	The results suggested that the programme was acceptable to health care staff because of the internal support it provided, the effective clinician leadership it featured, the fact that it took into account the internal culture and policies, its ability to foster a sense of ownership and the fact that it provided practical training in addition to the theory	+ +	+ + +

Relevance: + indicates low, +  + indicates moderate, +  +  + indicates high

Rigour: + indicates low, +  + indicates moderate, +  +  + indicates high

### Description of the educational interventions

**Fig. 3:**
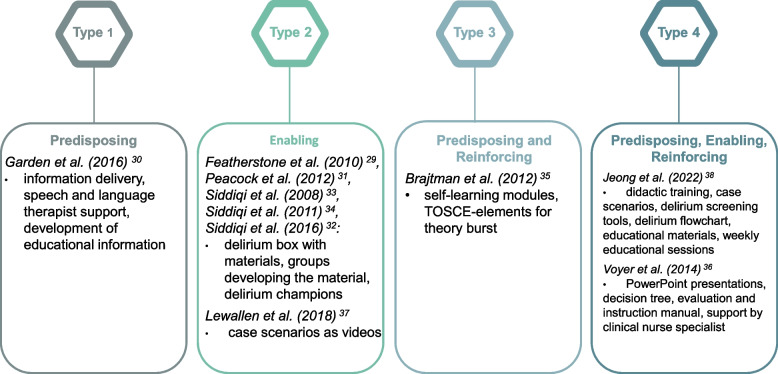
Classification of the publications included in this review according to the PRECEDE model (own illustration) [39]

Due to the diverse types of design employed in educational interventions, this realist review uses the Predisposing, Reinforcing and Enabling Constructs in Educational Diagnosis and Evaluation (PRECEDE) model to systematise the educational interventions drawn from the included publications [39] (see Fig. 3). This model, which originated in the context of continuing medical education, assigns interventions to one of four types: Type 1 – predisposing factors (dissemination of information, communication, and didactics); Type 2 – predisposing factors and enabling strategies (facilitation of desired performance change, e.g., by using protocols and guidelines or by providing resources); Type 3 – predisposing and reinforcing factors (reinforcement of learning through reminders and feedback from peers and experts); and Type 4 – a single, multifaceted intervention or a combination of all three types of interventions.

We categorised the intervention investigated by Garden et al. as Type 1 (Predisposing) [30]. This intervention contained information delivery as well as support from a speech and language therapist. Additionally, educational information was developed by the participants in the publication. The study was oriented on the *Stop Delirium!* Intervention, but structured in a reduced way, as described in the following. We categorised six publications as Type 2 (Enabling) [29, 31–34, 37]. Five of these publications referred to the intervention *Stop delirium!* [29, 31–34]. This intervention is characterised by a training package that includes three twenty-minute sessions. In these flexible, interactive sessions, a delirium practitioner provides knowledge about delirium using a variety of written materials. In addition, facilities designate delirium champions who are available to answer questions concerning delirium. The *Stop Delirium!* intervention also includes a so-called delirium box. This box contains materials that were developed during the project by working groups in the facilities. The box thus serves as a resource for ongoing learning based on the use of checklists and care pathways. These customised materials were developed by the groups for their own facilities but can also be used across groups. In this context, delirium champions serve as supporters and contact persons within the facilities. In one publication, an educational intervention based on *Stop Delirium!* was used in a reduced form [30]. Another study was also classified as Type 2. The educational intervention included in this publication featured educational sessions as well as case scenarios in the form of videos [37].

We classified one study [35] as Type 3 (Predisposing and Reinforcing). The educational intervention reported in this publication was based on self-learning modules. Additionally, team-observed structured clinical encounter elements and a theory burst were used.

Type 4 (Predisposing, Enabling, Reinforcing) combines all three factors, and two publications included in the review were associated with this type [36, 38]. In the study conducted by Jeong et al. [38], predisposing factors were shown to include a combination of didactic training with related delirium case scenarios observed among patients in nursing homes, which were used to enhance the participants' understanding. Enabling factors were shown to include delirium screening tools, a delirium care flowchart and educational materials that can be implemented in clinical practice. Reinforcing factors were discussed with regard to the intervention group at the end of each educational session on a weekly basis. The educational intervention reported in the study conducted by Voyer et al. [36] was shaped by a multicomponent intervention consisting of PowerPoint presentations, a decision tree and an evaluation and intervention instruction manual. Additionally, a long-term-care clinical nurse specialist provided support for nurses in educational interventions.

### Context-mechanism-outcome configurations (CMOcs) identified in the individual publications

Nine of the included publications reported educational interventions that were targeted solely at nurses [29–34, 36–38]. One publication focused on interprofessional teamwork, in which context nurses were included on teams alongside members of other professional groups [35]. The corresponding CMOcs are shown in Table 2.

**Table 2:** Contexts, interventions, mechanisms and outcomes identified in the included publications

**Author(s) and year**	Context-mechanism-outcome configurations
Garden et al. (2016) [30]	When the staff at a nursing home where some residents are in the final phase of their lives, and which has effective management supported by initial funding and personal support from local stakeholders [C], receive an educational intervention [I], they feel more confident [M] resulting in empowered care home staff [O1], who are characterised by confidence in their ability to recognise, prevent, and manage factors associated with delirium, as well as their knowledge of those factors [O2]
Featherstone et al. (2010) [29]	Management and funding [C] Perception of the education as well spend time and a sustainable form of education in the longer term [O] When trained and untrained staff who exhibited a variety of levels of knowledge regarding delirium [C] receive an educational intervention about delirium based on case studies and "How would you feel?" cards [I], they develop a sense that they know the needs of residents and understand residents’ behaviour [M], which results in increased staff knowledge followed by the establishment of a delirium-sensitive environment and staff-empowerment [O] When nursing home staff [C] participate in the participatory design of a delirium prevention programme [I] they develop a sense of ownership and pride [M] that causes these tools to be powerful in practice When delirium practitioners [C] support an educational intervention targeting staff in nursing homes [I] by lowering barriers to the organisation of sessions [M], staff members feel empowered, and the implementation of practice changes is promoted [O]
Lewallen et al. (2019) [37]	Providing “hands-on-training” [M] When nurses with extensive experience in caring for older adults [C] experience ongoing support [I1] and education from a nurse who exhibits competence in the field of delirium [I2], they develop a sense of recognition [M] that results in awareness of delirium [O] When nurses who have extensive experience in caring for older adults [C] receive education sessions regarding the process of screening for delirium [I], they feel confident in their ability to assess delirium [M], which results in improvements in their delirium knowledge and skills [O]
Peacock et al. (2012) [31]	When nursing home staff who know their residents well [C] receive a complex educational intervention pertaining to delirium [I], they develop curiosity with regard to behaviour changes [M], which results in the ability to identify possible delirium episodes and manage delirium care [O] When nursing home staff working at a nursing home who know their residents well [C] receive a complex educational intervention pertaining to delirium [I] they recognise the importance of preventing delirium to ensure resident well-being [M], which is followed by improvements in communication and teamwork with respect to prevention of delirium [O] When nursing home staff with existing knowledge of delirium triggers [C] receive a complex form of education [I], they are able to use their observational skills in combination with their knowledge of triggers [M] to detect delirium based on changes in resident behaviour and to manage the underlying cause [O]
Siddiqi et al. (2016) [32]	Scientists investigating delirium itself without staff [C] High staff turnover [C] results in decreased ability to recognise the fluctuations that occur in delirium throughout the day [O] High prevalence of dementia and cognitive impairment [C] results in challenges to the detection of delirium even among experienced nursing home staff [O] When nursing home staff that exhibit high staff turnover rates [C1] as well as limited information handover between shifts [C2] participate in an educational intervention involving working groups and delirium champions [I] they miss the sense that they know the needs of residents and understanding residents’ behaviour [M], resulting in an incapacity to improve delirium care [O]
Siddiqi et al. (2008) [33]	Management support [C] Knowledge about residents [C] When nursing home staff characterised by poor communication among colleagues [C] receive a multicomponent educational intervention pertaining to delirium [I], they become aware of the communication problem [M] and understand its relevance with regard to delirium care [O] When nursing home staff who are receptive to training [C] participate in an educational intervention aimed at developing material on delirium care [I], they develop a sense of ownership and pride [M], which result in acceptance of the delirium intervention [O] When staff from nursing homes [C] receive an educational intervention using working groups and delirium champions and discuss target areas to improve delirium care [I] their interest is captured [M1] and their learning needs are met [M2] and perceive the time well spent [O]
Siddiqi et al. (2011) [34]	Nurses are familiar with states of confusion in everyday care [C] High proportion of staff who lack training [C] High staff turnover [C] Increase in recorded delirium episodes [O] High acceptability and intrinsic motivation [O] When nursing home staff who lack confidence regarding delirium management [C] receive an educational intervention involving working groups and delirium champions [I], they feel pride in being asked for their expertise [M1] and empowered [M2], a situation which results in increased awareness of delirium and an increase in staff self-reported confidence with regard to delirium care [O] When nursing home staff [C] receive an educational intervention involving working groups and delirium champions based on a participatory design [I], they experience the content relevant to their work [M], which results in high acceptability and intrinsic motivation to provide delirium care [O]
Brajtman et al. (2012) [35]	When an interprofessional team in a nursing home [C] participates in a multicomponent intervention [I] they develop a sense of belonging to the team [M] resulting in an improvement of knowledge and competence for delirium at the end of life [O] When an interprofessional team in a nursing home [C] receives an educational intervention about end-of-life delirium and interprofessional teamwork [I] it accepts the fact that delirium is an interprofessional challenge that requires team treatment [M] resulting in an improvement of interprofessional competence [O]
Jeong et al. (2022) [38]	When experienced nursing home staff [C] take part in a multicomponent study on delirium [I] it improves their confidence providing delirium care [M] resulting in improved knowledge regarding delirium and improved confidence when providing delirium care [O] When experienced nursing home staff [C] take part in a multicomponent study on delirium [I] and discuss real case delirium scenarios in a group and report their experiences with delirium care they develop awareness for delirium [M] resulting in an improved understanding of distinct clinical features of delirium [O]
Voyer et al. (2014) [36]	When experienced nursing home staff from one nursing home [C1] supported by a management to take part [C2] in the participatory development of a delirium prevention program [I] they develop a sense of ownership [M] resulting in an acceptance for the delirium prevention program [O] When nurses responsible for conducting an educational intervention in their nursing home [C] do not adhere to the specific time limit and content or do not provide all components [I] it is not possible to evaluate sessions or provide proper information [M] leading to difficulties applying learned knowledge and using tools [O]

[C]: context, [I]: intervention, [M]: mechanism, [O]: outcome

Supplemented by numbering (e.g. [C1]) if several of the parameters (in this case context factors) come together in a CMOc

### Merged CMOcs

**Fig. 4:**
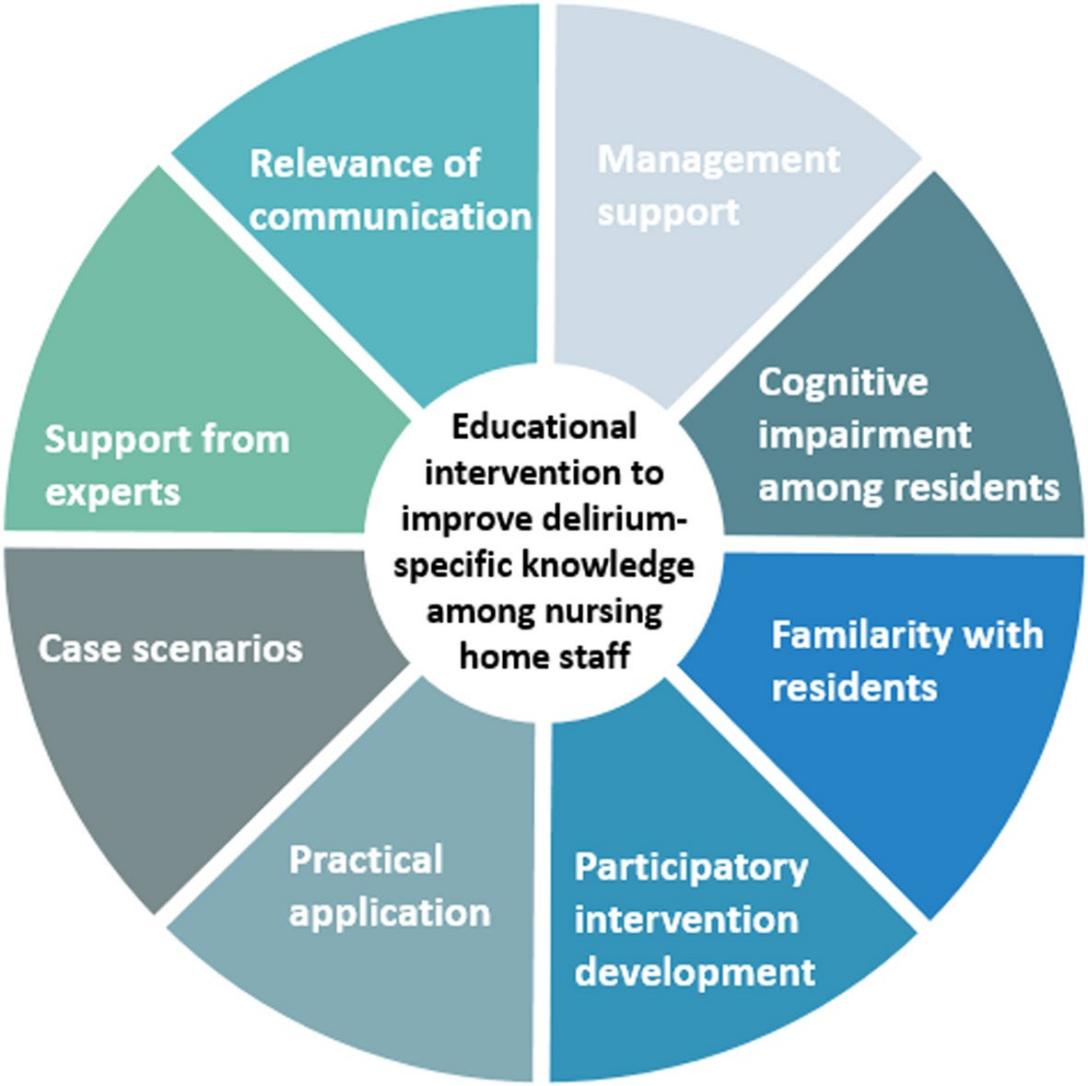
Initial programme theory on educational interventions aimed at improving delirium-specific knowledge among nursing home staff (authors’ own illustration)

The components of the initial programme theory are the synthesised CMOcs (see Fig. 4). The combined CMOcs are based on the CMOcs identified in the individual publications.

### Management support

We observed that support for management in the nursing home determines whether the educational intervention is successful [29, 30, 33, 34, 36]. We formulated the following CMOc by combining the findings reported in the included publications:

*When nursing home management supports (does not support) nursing home staff in the task of implementing educational interventions aimed at promoting delirium-specific expertise, the barriers faced by nursing home staff that lead them to accept (reject) the educational intervention and change (avoid changing) their behaviour are broken down (built up)* [29, 30, 33, 34, 36].

Management support with regard to the implementation of educational interventions enables nursing home staff to use resources (e.g., time resources or shift scheduling) to address the phenomenon of delirium [29]. Financial support provided by external sources can support the establishment of free spaces and the integration of educational interventions in a sustainable way [30, 34]. In the discussions that occurred during the development of the realist review, stakeholders also considered management to be a fundamental component of educational interventions in general.

### Cognitive impairment among residents

One reason why the implementation of educational interventions targeting delirium may fail pertains to the high number of residents who exhibit severe cognitive impairments and the complexity of detecting delirium among this group [31, 32]. The following CMOc was formulated for this purpose:

*A high prevalence of (severe) cognitive impairment among residents from nursing homes may lead to unsuccessful educational interventions targeting nursing home staff, particularly because the detection of delirium among residents with severe cognitive impairment is a major challenge* [31, 32].

One challenge in this context pertains to the detection of delirium in the target group. Even for experienced nursing home staff, detecting delirium in residents who exhibit severe cognitive impairment can be challenging [31].

In the exchanges with stakeholders, it became clear that recognising hypoactive delirium, which is distinct from hyperactive delirium, is also particularly challenging. This distinction is often only possible based on an extensive interview or medical history.

### Familiarity with residents

We assumed that the nursing home staff members’ knowledge of and familiarity with the residents is highly important [32, 33].

*If nursing home staff members (do not) know the residents in nursing homes in which they work, educational interventions aimed at promoting delirium-specific expertise can be successful (unsuccessful) because these staff members can (cannot) identify the behavioural changes that are characteristic of delirium due to their (lack of) familiarity with the residents; accordingly, delirium-sensitive care can (cannot) be improved* [32, 33].

It became clear in the publications that challenges arise with regard to recognising the symptoms of delirium due to such a lack of familiarity [32, 33]. Behavioural abnormalities and changes cannot be perceived if the typical behaviour of residents is unknown. This issue arose in the included publications as a result of high degrees of staff turnover [32, 33]. In this case, educational interventions aimed at promoting delirium-specific expertise remain ineffective [32, 33].

In addition, the review revealed that knowing the residents well is closely linked to the development of curiosity about behavioural changes, which results in an improved ability to identify delirium [31]. This reference to familiarity is closely linked to the realisation that knowledge of the needs of residents leads to the development of a delirium-sensitive environment [29].

The stakeholders confirmed our assumption that it is common for nursing home staff to notice that something is wrong; however, no structured approach to the assessment of delirium is available. Nurses are familiar with the progression of this phenomenon and can therefore be well equipped to differentiate between dementia and delirium.

### Participatory intervention development

Involving nursing home staff in the development of educational interventions in planning and implementation can promote their subsequent acceptance of the intervention due to their development of a sense of ownership [29, 33, 36] and pride [29, 34]. The integration of nursing home staff can therefore facilitate the development of tailored interventions [29, 33, 34, 36].

*When nursing home staff are involved in the development of educational interventions aimed at promoting delirium-specific knowledge, they experience a sense of pride, thus increasing their acceptance of and interest in the intervention* [29, 33, 34, 36].

The integration of nursing home staff is also helpful because they have essential information about barriers to the organisation of sessions, which can thereby be lowered [29].

### Practical application

It was particularly clear that educational interventions aimed at promoting delirium-specific expertise that also provided opportunities to engage in hands-on activities were helpful with regard to the application of theoretical knowledge. A sole focus on theoretical knowledge is not effective with regard to the application of the newly acquired knowledge in everyday life [36–38].

*When nursing home staff have the opportunity to apply the knowledge they have learned practically through educational interventions, their learning needs are addressed in a way that enables them to become more confident and self-efficient with regard to the provision of delirium-sensitive care* [36–38].

Educational interventions that provide the opportunity to engage at a hands-on level and directly apply one’s knowledge to enhance one’ skills and actively learn can be promising in this respect [37].

From the stakeholder discussion it was apparent that although learning opportunities can raise awareness, the topic of delirium is complex and thus involves barriers to active practice and implementation. It was also mentioned that real-life situations from health care are much more memorable and less likely to be forgotten than purely theoretical content. One nurse mentioned that a skills lab experience could also be helpful here.

### Case scenarios

Combining educational interventions that include practical components and lead nursing home staff through case scenarios in discussions can provide increased confidence in delirium-sensitive care [38].

In five publications, positive results were obtained by working with case scenarios [29, 31, 35, 37, 38]. The presentation of case scenarios in this context ranged from purely written accounts [29, 31, 35, 38] to videos.

*When nursing home staff have the opportunity to share and discuss their experiences using case scenarios in the context of educational interventions aimed at promoting delirium-specific expertise, they can improve their understanding of the clinical expression and relevance of delirium in their daily work and thereby provide more delirium-sensitive care* [29, 31, 35, 37, 38].

Working with case scenarios was experienced in the publications as very enriching with regard to the exchange of experiences involving comparable situations [38]. Brajtman et al. further noted that such exchange is conducive to interprofessional work and understanding [35]. An understanding of the clinical manifestations of delirium that is facilitated in this manner may be particularly helpful. According to Featherstone et al., case studies can help individuals relate educational interventions to their daily work and identify parallels [29].

Case studies in the context of learning opportunities were also cited as helpful in discussions with stakeholders. In addition, videos were viewed as helpful, and it was noted that texts should be rather short. It was also noted that discussions and exchanges can often convey more knowledge than pure information input. Case scenarios can certainly provide support in this context.

### Support from experts

Individual publications have shown that it is crucial to ensure that people who have rich experience or specific training are available as experts [29, 37].

*When nursing home staff are supported by delirium experts in the context of educational interventions, these experts can serve as role models and reduce barriers to ensure that participants’ learning needs can be addressed individually and that the training is accepted, thereby contributing to the improvement of their knowledge* [29, 37].

Nursing home staff benefit from the training they receive from experts in delirium [32–34]. The decisive factor in the publications was that the persons in question were accompanied throughout a more extensive process and received ongoing support [37]. Furthermore, the regularity of the training sessions was crucial. In addition, experienced delirium practitioners can detect the barriers that arise in the context of educational interventions and help remove them [29].

### Relevance of communication

Communication among nursing home staff plays an essential role in the prevention, diagnosis and treatment of delirium. Therefore, educational interventions aimed at promoting delirium-specific knowledge that focus on communication among nursing home staff can have positive impacts [29, 31–33]. Relevant possibilities regarding structured handovers with sufficient time can enable risk profiles and behavioural changes to be communicated and thus enable the fluctuating course that is characteristic of delirium to be identified [32].

*When nursing home staff understand that communication concerning behavioural changes and existing risk factors for the development of delirium among residents is highly relevant, they become aware of the importance of communication and their own roles, thus enabling them to provide delirium-sensitive care* [29, 31–33].

The exchanges with stakeholders highlighted the relevance of integrating the term “delirium” into communication. The use of other terminology may trivialise delirium and thus decrease nurses' awareness of the syndrome, which is associated with numerous negative outcomes.

## Discussion

The aim of this realist review was to develop an initial programme theory to determine how, why and under what conditions educational interventions aimed at promoting delirium-specific knowledge among health care professionals in nursing homes work. In the following, the theory is summarized once again, compared with the findings of implementation research and individual aspects are discussed in more detail.

### Initial programme theory

The initial programme theory, which is based on the theory of situated learning, suggests that the following factors have impacts in this context: (1) management support, (2) cognitive impairment among residents, (3) familiarity with residents, (4) participatory intervention development, (5) practical application, (6) case scenarios, (7) support from experts, and (8) relevance of communication. They can be divided into two groups concerning their focus on the context (factors 1–3) and the intervention (factors 4–8).

### Consistency with implementation science

Some of the results observed are consistent with the findings of implementation research [40]. In addition to structural support from management, the involvement of delirium experts, who can serve as role models for nursing staff, and participatory and practical approaches have been shown to support nursing staff in the development and implementation of an educational intervention on delirium in nursing homes. Support from management and experts enables health care professionals to develop sufficient confidence to act competently with regard to the detection and treatment of delirium [41].

### Impact on delirium superimposed on dementia

As mentioned beforehand, it is crucial to consider the fact that residents with existing (severe) cognitive impairment are at more risk of developing delirium than are residents without cognitive impairment. It is therefore crucial to provide specific support here. Particularly in regard to DSD, the recognition of delirium has proven to constitute a special challenge, such that even educational interventions may not be sufficient to improve the current situation. Although diagnostic tests can be used to detect DSD, validation publications remain very sparse, and these tests are characterised by an insufficient level of diagnostic quality [42]. The involvement of relatives could be an option for assessing the condition of a resident with suspected DSD. Due to the fact that the majority of nursing home residents are affected by (severe) cognitive impairments and are getting older, the proportion of delirium will also increase in the future [43]. In this context, the risk of confusion between dementia and delirium must also be taken into account. This target group should be given special consideration in the context of educational interventions on delirium in nursing homes.

### Considerations on staff shortages

This point is followed by another aspect of initial programme theory, which refers to the degree of familiarity of nursing home staff members with residents. Nursing home staff who know their residents will have fewer problems recognising and then intervening in sudden changes in behaviour; thus, residents who are particularly at risk for delirium should be cared for by nursing home staff who know them well [21]. However, staff turnover in nursing homes can exacerbate this lack of familiarity. Accordingly, from a delirium prevention perspective, consideration should be given to ways of limiting staff turnover and to ensure that delirium is assessed by nursing home staff who know the residents.

### Strengthening sensitivity regarding delirium in nursing homes

Moreover, educational interventions should also focus on communication. It is important to ensure that high-risk profiles are described and that behavioural changes are relayed during shift handoffs to provide an overview of delirium (risk) [21]. If delirium is understood as an interprofessional problem, the focus is not only on exchanges between shifts but also extends beyond the boundaries of the profession. It is therefore very important to use the term delirium in everyday nursing care and to avoid using terms that tend to obscure the existing emergency situation, which can also cause interprofessional communication to be impaired [44].

We know from discussions with stakeholders that delirium is a familiar phenomenon in hospitals, whereas in nursing homes, the term delirium is not even known or used by many nurses. Publications on existing knowledge of delirium in nursing homes remain scarce, but figures drawn from other settings, such as hospitals or hospice, support the claim that nurses' knowledge of delirium is limited [45, 46], although hospitals feature at least some awareness of the importance of delirium knowledge [47]. Educational interventions aimed at enhancing the delirium-specific knowledge of health care professionals in nursing homes are therefore highly important with regard to raising awareness of this topic.

If people are not aware of or vigilant with regard to the phenomenon of delirium in nursing homes, this gap can represent a major barrier. This conclusion can be viewed as a key finding of the realist review. Nursing staff must be trained accordingly so that they have the necessary self-confidence and an open attitude towards the phenomenon to play an active role in preventing and recognizing delirium in terms of knowing, meaning and doing [48]. A delirium-sensitive culture in nursing homes can support sensitivity [49], and it is helpful if the phenomenon is not misjudged during the course of the strenuous daily routines and if hospitalisations can be prevented.

### Didactic implementation

It is evident that educational interventions aimed at promoting delirium-specific knowledge should include interactive elements [50]. In other settings, for example, simulation-based education [51, 52] or the use of serious games [53] have already been identified as innovative approaches to the task of increasing delirium-specific knowledge. The initial programme theory developed in this context will be further refined in the future and will serve as a basis for the development of complex interventions aimed at the prevention of delirium in nursing homes.

### Strengths and limitations

Since we found nine publications on educational interventions for nurses and only one publication on a general multiprofessional educational intervention, we assume that the education and training of other professional groups and additional multiprofessional approaches are currently at an insufficient stage of development. The development of such approaches may be helpful with regard to the initial programme theory included in this review.

A strength of this review pertained to the discussions with the stakeholders, which enabled the study team to reflect on their theoretical assumptions and interpret their findings.

Because no standardised procedure was available for the inclusion of stakeholders, we described this phase of the review in detail so it can be used as a blueprint for potential further realist reviews. The inclusion of stakeholders also enabled the review to be closely related to practice as a result of the constant comparisons between findings from the literature and practice. In addition, the researchers were sensitised to particular challenges in practice through such exchange, thus enabling them to detect these issues in the publications and to interpret them in an informed manner. A discrepancy between the included publications and the experiences of the stakeholders can be noted in this context. While the publications included in this review, which were almost exclusively conducted in the Anglo-American region, showed that delirium care is recognised as an integral part of nursing in nursing homes, this fact does not seem to be taken for granted in the German context. One possible reason for this difference is the structural neglect of the phenomenon of delirium in nursing homes and the associated dominant view of delirium, which depicts it as a hospital-specific or specifically intensive care phenomenon.

One critical point is that no grey literature could be obtained in this review. Due to the unstructured method used for the grey literature search on account of project-related time constraints, it was unfortunately not possible to find publications that met the inclusion criteria. An iterative process that involves searching, analysing, searching, and analysing could also not be used due to time constraints. Nonetheless, the literature analysis was based on an iterative process, although it contained only the literature that was identified in the systematic search.

Finally, it should be mentioned that the majority of the publications focused on the Stop Delirium! intervention. This is crucial to consider, as a procedure has been implemented in a similar way in several studies and thus extensive information is available on this approach.

## Conclusion

Educational interventions aimed at promoting delirium-specific expertise among nursing home staff should be characterised by methodological diversity if they are to be sufficiently sensitive to the clinical manifestations of delirium. Nursing home staff members are fundamentally responsible for identifying delirium in vulnerable residents and thus detecting medical emergencies associated with numerous negative outcomes. The targeted promotion of delirium-specific knowledge must therefore be emphasised in the context of training and continuing education.

### Supplementary Information

Supplementary Material 1.

Supplementary Material 2.

Supplementary Material 3.

## Data Availability

The datasets used and analysed during the realist review are available from the corresponding author on reasonable request.
