# Supplementary material for: Educational interventions aimed at improving knowledge of delirium among nursing home staff—a realist review
Source: BMC Geriatr. 2024 Jul 25;24:633. doi: 10.1186/s12877-024-05213-9 (PMC11274774; doi:10.1186/s12877-024-05213-9)
Supplement: Supplementary file 2 — Supplementary Material 2. [file 12877_2024_5213_MOESM2_ESM.docx]

Appendix 1: Search strategy

**Search strategy PubMed**

| **No** | **Syntax** | **Hits** |
| --- | --- | --- |
| **#1** | (Delirium [Mesh]  OR delir* [Title/Abstract]  OR Delir [Title/Abstract]  OR Verwirr* [Title/Abstract]  OR acute confusion* [Title/Abstract]  OR organic brain syndrome [Title/Abstract]  OR acute organic psychosyndrome [Title/Abstract]  OR acute organic brain syndrome [Title/Abstract]) | 23 095 |
| **#2** | (Langzeitpfleg* [Title/Abstract]  OR Pflegeheim [Title/Abstract]  OR longterm care [Title/Abstract]  OR long term care [Title/Abstract]  OR ltc [Title/Abstract]  OR care home [Title/Abstract]  OR care facility [Title/Abstract]  OR care facilities [Title/Abstract]  OR nursing facility [Title/Abstract]  OR nursing facilities [Title/Abstract]  OR residential home [Title/Abstract]  OR residential homes [Title/Abstract]  OR residential care [Title/Abstract]  OR nursing home [Title/Abstract]  OR residential facility [Title/Abstract]  OR home for the elderly [Title/Abstract]  OR residential facilities [Mesh]  OR Long-Term Care [Mesh]  OR Homes for the Aged [Mesh]  OR Nursing Homes [Mesh]) | 120 592 |
| **#1 AND #2** | ((Langzeitpfleg* [Title/Abstract]  OR Pflegeheim [Title/Abstract]  OR longterm care [Title/Abstract]  OR long term care [Title/Abstract]  OR ltc [Title/Abstract]  OR care home [Title/Abstract]  OR care facility [Title/Abstract]  OR care facilities [Title/Abstract]  OR nursing facility [Title/Abstract]  OR nursing facilities [Title/Abstract]  OR residential home [Title/Abstract]  OR residential homes [Title/Abstract]  OR residential care [Title/Abstract]  OR nursing home [Title/Abstract]  OR residential facility [Title/Abstract]  OR home for the elderly [Title/Abstract]  OR residential facilities [Mesh]  OR Long-Term Care [Mesh]  OR Homes for the Aged [Mesh]  OR Nursing Homes [Mesh]))  AND  ((Delirium [Mesh]  OR delir* [Title/Abstract]  OR Delir [Title/Abstract]  OR Verwirr* [Title/Abstract]  OR acute confusion* [Title/Abstract]  OR organic brain syndrome [Title/Abstract]  OR acute organic brain syndrome [Title/Abstract])) | 860 |
| **#3** | (educat*[Title/Abstract]  OR intervention*[Title/Abstract]  OR program[Title/Abstract]  OR training[Title/Abstract]  OR Schulung [Title/Abstract]  OR learning [Title/Abstract]  OR knowledge [Title/Abstract]  OR Wissen* [Title/Abstract] | 3 789 291 |
| **#1 AND #2 AND #3** | (Delirium [Mesh]  OR delir* [Title/Abstract]  OR Delir [Title/Abstract]  OR Verwirr* [Title/Abstract]  OR acute confusion* [Title/Abstract]  OR organic brain syndrome  [Title/Abstract]  OR acute organic brain syndrome [Title/Abstract])  AND  (Langzeitpfleg* [Title/Abstract]  OR Pflegeheim [Title/Abstract]  OR longterm care [Title/Abstract]  OR long term care [Title/Abstract]  OR ltc [Title/Abstract]  OR care home [Title/Abstract]  OR care facility [Title/Abstract]  OR care facilities [Title/Abstract]  OR nursing facility [Title/Abstract]  OR nursing facilities [Title/Abstract]  OR residential home [Title/Abstract]  OR residential homes [Title/Abstract]  OR residential care [Title/Abstract]  OR nursing home [Title/Abstract]  OR residential facility [Title/Abstract]  OR home for the elderly [Title/Abstract]  OR residential facilities [Mesh]  OR Long-Term Care [Mesh]  OR Homes for the Aged [Mesh]  OR Nursing Homes [Mesh])  AND  (educat*[Title/Abstract]  OR intervention*[Title/Abstract]  OR program[Title/Abstract]  OR training[Title/Abstract]  OR Schulung [Title/Abstract]  OR learning [Title/Abstract]  OR knowledge [Title/Abstract]  OR Wissen* [Title/Abstract]) | 287 |

**Suchstrategie CINAHL**

| **No** | **Syntax** | **Hits** |
| --- | --- | --- |
| **#1** | (AB delir*  OR AB acute confusion*  OR AB Verwirrtheit  OR AB organic brain syndrome  OR AB acute organic brain syndrome  OR (TI delir*  OR TI acute confusion*  OR TI Verwirrtheit  OR TI organic brain syndrome  OR TI acute organic brain syndrome)  OR (MH "Delirium")  OR (MH "Delirium Management (Iowa NIC)") ) | 38 717 |
| **#2** | (TI Langzeitpfleg*  OR TI Pflegeheim  OR TI longterm care  OR (MH "Long Term Care")  OR TI long term care  OR TI ltc  OR TI care home  OR TI care facilit*  OR TI nursing facilit*  OR TI residential home  OR TI residential homes  OR TI residential care  OR TI nursing home  OR (MH "Nursing Homes")  OR TI residential facility  OR TI residential facilities  OR (MH "Residential Facilities")  OR (AB Langzeitpfleg*  OR AB Pflegeheim  OR AB longterm care  OR AB long term care  OR AB ltc  OR AB care home  OR AB care facilit*  OR AB nursing facilit*  OR AB residential home  OR AB residential homes  OR AB residential care  OR AB nursing home  OR AB residential facility  OR AB residential facilities | 401 242 |
| **#1 AND #2** | ( (AB delir*  OR AB acute confusion*  OR AB Verwirrtheit  OR AB organic brain syndrome  OR AB acute organic brain syndrome)  OR (TI delir* OR TI acute confusion*  OR TI Verwirrtheit  OR TI organic brain syndrome  OR TI acute organic brain syndrome)  OR ( (MH "Delirium")  OR (MH "Delirium Management (Iowa NIC)") ) )  AND  (TI Langzeitpfleg*  OR TI Pflegeheim  OR TI longterm care  OR (MH "Long Term Care")  OR TI long term care  OR TI ltc OR TI care home  OR TI care facilit*  OR TI nursing facilit*  OR TI residential home  OR TI residential homes  OR TI residential care  OR TI nursing home  OR (MH "Nursing Homes")  OR TI residential facility  OR TI residential facilities  OR (MH "Residential Facilities")  OR TI home for the elderly)  OR (AB Langzeitpfleg*  OR AB Pflegeheim  OR AB longterm care  OR AB long term care  OR AB ltc OR AB care home  OR AB care facilit*  OR AB nursing facilit*  OR AB residential home  OR AB residential homes  OR AB residential care  OR AB nursing home  OR AB residential facility  OR AB residential facilities  OR (AB home for the elderly) ) | 2 153 |
| **#3** | ( TI educat*  OR TI intervention*  OR TI program  OR TI training  OR TI Schulung  OR TI learning  OR TI knowledge  OR TI Wissen  OR AB educat*  OR AB intervention*  OR AB program  OR AB training  OR AB Schulung  OR AB learning  OR AB knowledge  OR AB Wissen) | 220487 |
| **#1 AND #2 AND #3** | (((AB delir*  OR AB acute confusion*  OR AB Verwirrtheit  OR AB organic brain syndrome  OR AB acute organic brain syndrome)  OR (TI delir* OR TI acute confusion*  OR TI Verwirrtheit  OR TI organic brain syndrome  OR TI acute organic brain syndrome)  OR ( (MH "Delirium")  OR (MH "Delirium Management (Iowa NIC)") ) )  AND  (TI Langzeitpfleg*  OR TI Pflegeheim  OR TI longterm care  OR (MH "Long Term Care")  OR TI long term care  OR TI ltc  OR TI care home  OR TI care facilit*  OR TI nursing facilit*  OR TI residential home  OR TI residential homes  OR TI residential care  OR TI nursing home  OR (MH "Nursing Homes")  OR TI residential facility  OR TI residential facilities  OR (MH "Residential Facilities")  OR TI home for the elderly)  OR (AB Langzeitpfleg*  OR AB Pflegeheim  OR AB longterm care  OR AB long term care  OR AB ltc OR AB care home  OR AB care facilit*  OR AB nursing facilit*  OR AB residential home  OR AB residential homes  OR AB residential care  OR AB nursing home  OR AB residential facility  OR AB residential facilities  OR AB home for the elderly)  AND  ( TI educat*  OR TI intervention*  OR TI program  OR TI training  OR TI Schulung  OR TI learning  OR TI knowledge  OR TI Wissen  OR AB educat*  OR AB intervention*  OR AB program  OR AB training  OR AB Schulung  OR AB learning  OR AB knowledge  OR AB Wissen) | 608 |

**Search strategy Scopus**

| **No** | **Syntax** | **Hits** |
| --- | --- | --- |
| **#1** | ( TITLE-ABS-KEY ( delirium )  OR TITLE-ABS-KEY ( delir* )  OR TITLE-ABS-KEY ( verwirr* )  OR TITLE-ABS-KEY ( acute AND confusion* )  OR TITLE-ABS-KEY ( {organic brain syndrome} )  OR TITLE-ABS-KEY ( {acute organic psychosyndrome} )  OR TITLE-ABS-KEY ( {acute organic brain syndrome} ) ) | 57 915 |
| **#2** | ( TITLE-ABS-KEY ( langzeitpfleg* )  OR TITLE-ABS-KEY ( pflegeheim )  OR TITLE-ABS-KEY ( {longterm care} )  OR TITLE-ABS-KEY ( {long term care} )  OR TITLE-ABS-KEY ( ltc )  OR TITLE-ABS-KEY ( {care home} )  OR TITLE-ABS-KEY ( {care facility} )  OR TITLE-ABS-KEY ( {care facilities} )  OR TITLE-ABS-KEY ( {nursing facility} )  OR TITLE-ABS-KEY ( {nursing facilities} )  OR TITLE-ABS-KEY ( {residential home} )  OR TITLE-ABS-KEY ( {residential homes} )  OR TITLE-ABS-KEY ( {residential care} )  OR TITLE-ABS-KEY ( {nursing home} )  OR TITLE-ABS-KEY ( {residential facility} )  OR TITLE-ABS-KEY ( {home for the elderly} )  OR TITLE-ABS-KEY ( {residential facilities} )  OR TITLE-ABS-KEY ( {Long-Term Care})  OR TITLE-ABS-KEY ( {Homes for the Aged} )  OR TITLE-ABS-KEY ( {Nursing Homes})  OR TITLE-ABS-KEY ( {Nursing Home}) ) | 359 222 |
| **#1 AND #2** | ( ( TITLE-ABS-KEY ( langzeitpfleg* )  OR TITLE-ABS-KEY ( pflegeheim )  OR TITLE-ABS-KEY ( {longterm care} )  OR TITLE-ABS-KEY ( {long term care} )  TITLE-ABS-KEY ( ltc )  OR TITLE-ABS-KEY ( {care home} )  OR TITLE-ABS-KEY ( {care facility} )  OR TITLE-ABS-KEY ( {care facilities} )  OR TITLE-ABS-KEY ( {nursing facility} )  OR TITLE-ABS-KEY ( {nursing facilities} )  OR TITLE-ABS-KEY ( {residential home} )  OR TITLE-ABS-KEY ( {residential homes} )  OR TITLE-ABS-KEY ( {residential care} )  OR TITLE-ABS-KEY ( {nursing home} )  OR TITLE-ABS-KEY ( {residential facility} )  OR TITLE-ABS-KEY ( {home for the elderly} )  OR TITLE-ABS-KEY ( {residential facilities} )  OR TITLE-ABS-KEY ( {Long-Term Care} )  OR TITLE-ABS-KEY ( {Homes for the Aged} )  OR TITLE-ABS-KEY ( {Nursing Homes})  OR TITLE-ABS-KEY ( {Nursing Home} ) ) )  AND ( ( TITLE-ABS-KEY ( delirium )  OR TITLE-ABS-KEY ( delir* )  OR TITLE-ABS-KEY ( verwirr* )  OR TITLE-ABS-KEY ( acute AND confusion* )  OR TITLE-ABS-KEY ( {organic brain syndrome} )  OR TITLE-ABS-KEY ( {acute organic psychosyndrome} )  OR TITLE-ABS-KEY ( {acute organic brain syndrome} ) ) ) | 2 366 |
| **#3** | ( TITLE-ABS-KEY ( educat* )  OR TITLE-ABS-KEY ( intervention* )  OR TITLE-ABS-KEY ( program )  OR TITLE-ABS-KEY ( training )  OR TITLE-ABS-KEY ( schulung )  OR TITLE-ABS-KEY ( learning )  OR TITLE-ABS-KEY ( knowledge )  OR TITLE-ABS-KEY ( wissen* ) ) | 10 404 473 |
| **#1 AND #2 AND #3** | ( ( TITLE-ABS-KEY ( delirium )  OR TITLE-ABS-KEY ( delir* )  OR TITLE-ABS-KEY ( verwirr* )  OR TITLE-ABS-KEY ( acute AND confusion* )  OR TITLE-ABS-KEY ( {organic brain syndrome} )  OR TITLE-ABS-KEY ( {acute organic psychosyndrome} )  OR TITLE-ABS-KEY ( {acute organic brain syndrome} ) ) )  AND ( ( TITLE-ABS-KEY ( langzeitpfleg* )  OR TITLE-ABS-KEY ( pflegeheim )  OR TITLE-ABS-KEY ( {longterm care} )  OR TITLE-ABS-KEY ( {long term care} )  OR TITLE-ABS-KEY ( ltc )  OR TITLE-ABS-KEY ( {care home} )  OR TITLE-ABS-KEY ( {care facility} )  OR TITLE-ABS-KEY ( {care facilities} )  OR TITLE-ABS-KEY ( {nursing facility} )  OR TITLE-ABS-KEY ( {nursing facilities} )  OR TITLE-ABS-KEY ( {residential home} )  OR TITLE-ABS-KEY ( {residential homes} )  OR TITLE-ABS-KEY ( {residential care})  OR TITLE-ABS-KEY ( {nursing home} )  OR TITLE-ABS-KEY ( {residential facility} )  OR TITLE-ABS-KEY ( {home for the elderly} )  OR TITLE-ABS-KEY ( {residential facilities} )  OR TITLE-ABS-KEY ( {Long-Term Care} )  OR TITLE-ABS-KEY ( {Homes for the Aged} )  OR TITLE-ABS-KEY ( {Nursing Homes} )  OR TITLE-ABS-KEY ( {Nursing Home} ) ) )  AND ( ( TITLE-ABS-KEY ( educat* )  OR TITLE-ABS-KEY ( intervention* )  OR TITLE-ABS-KEY ( program )  OR TITLE-ABS-KEY ( training )  OR TITLE-ABS-KEY ( schulung )  OR TITLE-ABS-KEY ( learning )  OR TITLE-ABS-KEY ( knowledge )  OR TITLE-ABS-KEY ( wissen* ) ) ) | 746 |

**Searchstrategy Web of Science**

| **No** | **Syntax** | **Hits** |
| --- | --- | --- |
| **#1** | (((((TI=(delirium))  OR TI=(delir*))  OR TI=(verwirr*))  OR TI=("acute confusion*"))  OR TI=("organic brain syndrome"))  OR TI=("acute organic psychosyndrome")  OR ((((((AB=(delirium))  OR AB=(delir*))  OR AB=(verwirr*))  OR AB=("acute confusion*"))  OR AB=("organic brain syndrome"))  OR AB=("acute organic psychosyndrome")) | 20 772 |
| **#2** | (((((((((((((((((((TI=(langzeitpfleg*))  OR TI=(pflegeheim))  OR TI=("longterm care"))  OR TI=("long term care"))  OR TI=(ltc))  OR TI=("care home"))  OR TI=("care facility"))  OR TI=("care facilites"))  OR TI=("nursing facility"))  OR TI=("nursing facilities"))  OR TI=("residental home"))  OR TI=("residental homes"))  OR TI=("residental care"))  OR TI=("nursing home"))  OR TI=("residental facility"))  OR TI=("home for the elderly"))  OR TI=("residental facilities"))  OR TI=("Long-term care"))  OR TI=("homes for the aged"))  OR TI=("nursing homes")  OR (((((((((((((((((((AB=(langzeitpfleg*))  OR AB=(pflegeheim))  OR AB=("longterm care"))  OR AB=("long term care"))  OR AB=(ltc))  OR AB=("care home"))  OR AB=("care facility"))  OR AB=("care facilites"))  OR AB=("nursing facility"))  OR AB=("nursing facilities"))  OR AB=("residental home"))  OR AB=("residental homes"))  OR AB=("residental care"))  OR AB=("nursing home"))  OR AB=("residental facility"))  OR AB=("home for the elderly"))  OR AB=("residental facilities"))  OR AB=("Long-term care"))  OR AB=("homes for the aged"))  OR AB=("nursing homes") | 66 460 |
| **#1 AND #2** | (((((TI=(delirium))  OR TI=(delir*))  OR TI=(verwirr*))  OR TI=("acute confusion*"))  OR TI=("organic brain syndrome"))  OR TI=("acute organic psychosyndrome")  OR ((((((AB=(delirium))  OR AB=(delir*))  OR AB=(verwirr*))  OR AB=("acute confusion*"))  OR AB=("organic brain syndrome"))  OR AB=("acute organic psychosyndrome"))  AND (((((((((((((((((((TI=(langzeitpfleg*))  OR TI=(pflegeheim))  OR TI=("longterm care"))  OR TI=("long term care"))  OR TI=(ltc))  OR TI=("care home"))  OR TI=("care facility"))  OR TI=("care facilites"))  OR TI=("nursing facility"))  OR TI=("nursing facilities"))  OR TI=("residental home"))  OR TI=("residental homes"))  OR TI=("residental care"))  OR TI=("nursing home"))  OR TI=("residental facility"))  OR TI=("home for the elderly"))  OR TI=("residental facilities"))  OR TI=("Long-term care"))  OR TI=("homes for the aged"))  OR TI=("nursing homes")  OR (((((((((((((((((((AB=(langzeitpfleg*))  OR AB=(pflegeheim))  OR AB=("longterm care"))  OR AB=("long term care"))  OR AB=(ltc))  OR AB=("care home"))  OR AB=("care facility"))  OR AB=("care facilites"))  OR AB=("nursing facility"))  OR AB=("nursing facilities"))  OR AB=("residental home"))  OR AB=("residental homes"))  OR AB=("residental care"))  OR AB=("nursing home"))  OR AB=("residental facility"))  OR AB=("home for the elderly"))  OR AB=("residental facilities"))  OR AB=("Long-term care"))  OR AB=("homes for the aged"))  OR AB=("nursing homes" | 678 |
| **#3** | (((((((TI=(educat*))  OR TI=(intervention*))  OR TI=(program))  OR TI=(training))  OR TI=(schulung))  OR TI=(learning))  OR TI=(knowledge))  OR TI=(wissen*) | 2 250 171 |
| **#1 AND #2 AND #3** | (((((TI=(delirium))  OR TI=(delir*))  OR TI=(verwirr*))  OR TI=("acute confusion*"))  OR TI=("organic brain syndrome"))  OR TI=("acute organic psychosyndrome")  OR ((((((AB=(delirium))  OR AB=(delir*))  OR AB=(verwirr*))  OR AB=("acute confusion*"))  OR AB=("organic brain syndrome"))  OR AB=("acute organic psychosyndrome"))  AND (((((((((((((((((((TI=(langzeitpfleg*))  OR TI=(pflegeheim))  OR TI=("longterm care"))  OR TI=("long term care"))  OR TI=(ltc))  OR TI=("care home"))  OR TI=("care facility"))  OR TI=("care facilites"))  OR TI=("nursing facility"))  OR TI=("nursing facilities"))  OR TI=("residental home"))  OR TI=("residental homes"))  OR TI=("residental care"))  OR TI=("nursing home"))  OR TI=("residental facility"))  OR TI=("home for the elderly"))  OR TI=("residental facilities"))  OR TI=("Long-term care"))  OR TI=("homes for the aged"))  OR TI=("nursing homes")  OR (((((((((((((((((((AB=(langzeitpfleg*))  OR AB=(pflegeheim))  OR AB=("longterm care"))  OR AB=("long term care"))  OR AB=(ltc))  OR AB=("care home"))  OR AB=("care facility"))  OR AB=("care facilites"))  OR AB=("nursing facility"))  OR AB=("nursing facilities"))  OR AB=("residental home"))  OR AB=("residental homes"))  OR AB=("residental care"))  OR AB=("nursing home"))  OR AB=("residental facility"))  OR AB=("home for the elderly"))  OR AB=("residental facilities"))  OR AB=("Long-term care"))  OR AB=("homes for the aged"))  OR AB=("nursing homes")  AND (((((((TI=(educat*))  OR TI=(intervention*))  OR TI=(program))  OR TI=(training))  OR TI=(schulung))  OR TI=(learning))  OR TI=(knowledge))  OR TI=(wissen*) | 60 |
