# Supplementary material for: Educational interventions aimed at improving knowledge of delirium among nursing home staff—a realist review
Source: BMC Geriatr. 2024 Jul 25;24:633. doi: 10.1186/s12877-024-05213-9 (PMC11274774; doi:10.1186/s12877-024-05213-9)
Supplement: Supplementary file 3 — Supplementary Material 3. [file 12877_2024_5213_MOESM3_ESM.docx]

Educational interventions to improve knowledge of delirium among nursing home staff - a realist review

Appendix 3: Inclusion and exclusion criteria

|  | Inclusion criteria | Exclusion criteria |
| --- | --- | --- |
| Language | - German - English | - Other languages |
| Study design | - Any design |  |
| Forms of publication | - Any form |  |
| Format | - Full text available |  |
| Publication period | - No specific time period. |  |
| Interventional focus | - (Educational) interventions   or   - Multicomponent interventions   aiming to improve delirium-specific knowledge of healthcare professionals (e.g. nurses, assistance professions, physicians) |  |
| Setting | - International nursing home setting including short-term care and 24h-care | - Hospital - Hospice - Outpatient care |
| Delirium form | - Delirium in any form (according to ICD-10 or DSM-V classifications) | - Alcohol-induced delirium |
